# Direct evidence for increased disease resistance in polyandrous broods exists only in eusocial Hymenoptera

**DOI:** 10.1186/s12862-021-01925-3

**Published:** 2021-10-20

**Authors:** D. M. Soper, A. K. E. Ekroth, M. J. F. Martins

**Affiliations:** 1 Department of Biology, University of Dallas, 1845 E. Northgate Dr., Irving, TX 75062 USA; 2 Department of Zoology, University of Oxford, 11a Mansfield Road, Oxford, OX1 3SZ UK; 3 Interdisciplinary Center for Archaeology and Evolution of Human Behaviour (ICArEHB), Faculdade de Ciências Humanas e Sociais, Universidade do Algarve, Campus de Gambelas, 8005-139 Faro, Portugal; 4 Department of Paleobiology, National Museum of Natural History, Smithsonian Institution, Washington, DC 20013-7012 USA

**Keywords:** Multiple mating, Genetic diversity, Parasites, Animal behavior, Evolution

## Abstract

**Background:**

The ‘genetic diversity’ hypothesis posits that polyandry evolved as a mechanism to increase genetic diversity within broods. One extension of this hypothesis is the ‘genetic diversity for disease resistance’ hypothesis (GDDRH). Originally designed for eusocial Hymenoptera, GDDRH states that polyandry will evolve as an effect of lower parasite prevalence in genetically variable broods. However, this hypothesis has been broadly applied to several other taxa. It is unclear how much empirical evidence supports GDDRH specifically, especially outside eusocial Hymenoptera.

**Results:**

This question was addressed by conducting a literature review and posteriorly conducting meta-analyses on the data available using Hedges’s *g*. The literature review found 10 direct and 32 indirect studies with both having a strong publication bias towards Hymenoptera. Two meta-analyses were conducted and both found increased polyandry (direct tests; *n* = 8, *g* = 0.2283, *p* =  < 0.0001) and genetic diversity generated by other mechanisms (indirect tests; *n* = 10, *g* = 0.21, *p* =  < 0.0001) reduced parasite load. A subsequent moderator analysis revealed that there were no differences among Orders, indicating there may be applicability outside of Hymenoptera. However, due to publication bias and low sample size we must exercise caution with these results.

**Conclusion:**

Despite the fact that the GDDRH was developed for Hymenoptera, it is frequently applied to other taxa. This study highlights the low amount of direct evidence supporting GDDRH, particularly outside of eusocial Hymenoptera. It calls for future research to address species that have high dispersal rates and contain mixes of solitary and communal nesting.

**Supplementary Information:**

The online version contains supplementary material available at 10.1186/s12862-021-01925-3.

## Background

Polyandry is common among a wide variety of animal taxa [3, 21, 22, 65, 71], and is an evolutionary conundrum: Why mate with more than one male when females can typically fertilize all eggs with a single mating? Over the last few decades, the impact of polyandry and its consequences have been investigated in a diverse number of evolutionary phenomena. Polyandry influences (and is affected by) mechanisms such as cryptic female choice, sexual conflict, parental care, and ecological parameters such as resource accumulation and competition (e.g., reviewed in [37, 53, 71]. However, the evolutionary causes (and maintenance) of polyandry are still being explored.

Hymenopterans are particularly useful to better understand why polyandry might evolve. Unlike insects in general, polyandry is much rarer among social insects [68], the prominence of polyandry in certain eusocial taxa, when cooperation implicates genetic relatedness, remains an open question [74]. Hymenoptera display a wide diversity of mating systems including both sexual and asexual modes of reproduction [36]. Sexual mating strategies include monogamy, polygyny (multiple queens), polyandry (multiple mating by a single queen), and hyperpolyandry (> 5 males) [12].

The hypotheses put forth to explain the evolution of polyandry in Hymenopterans are varied. Page [50] argued that multiple mating by honeybee queens is the result of their genic sex determination. This idea was countered by Hamilton [23] and Sherman et al. [61] where they laid out the ‘genetic diversity for disease resistance’ hypothesis (from here on GDDRH). The GDDRH assumes that increased genetic diversity via polyandry will reduce the likelihood of parasitic infection, given that nests (colonies) provide prime conditions for parasite transfer (see review Wilson-Rich et al. [78] for further hypotheses on defense against disease in social insects). Some empirical support for the GDDRH has been found within the eusocial Hymenopteran (e.g., [5, 69], but the GDDRH has been extended to non-Hymenopteran taxa (e.g. [10, 80], lending to its prominence as an explanatory mechanism for the evolution of polyandry beyond the Hymenopterans. Kraus and Page [35] expressed doubt that the GDDRH is a sufficient explanation and rejected its application to honeybees. Sherman et al. [62] countered their argument by pointing out that genic sex determination is present in both monandrous and polyandrous Hympenopteran species. Palmer and Oldryod [51] later regard a suite of ‘genetic variation’ (GV) hypotheses (reviewed in [33] as the only likely explanation for the evolution of polyandry in the genus *Apis*. The discussion is far from resolved with several factors potentially contributing to the selection for increased genetic diversity via polyandry in the group [71], including the haplo-diploid sex-determination system [48].

One of the outcomes of polyandry is the generation of higher levels of genetic diversity within broods compared to that of a monandrous pair [81]. Increased genetic diversity within a population has multiple benefits. Genetically diverse populations are more efficient at adapting to biotic and abiotic environmental changes compared to populations with low genetic diversity (e.g. [27, 28, 67]. High levels of genetic diversity can result in increased population homeostasis [49], and can reduce competition for optimal sex ratios [45, 55]. In addition, research in the field of the evolution of sexual reproduction highlighted the importance of high levels of genetic diversity in outweighing the costs of male production relative to asexuality. The Red Queen hypothesis states that sexual reproduction (male production) evolves and is maintained under host/parasite coevolution. Males contribute by increasing the level of genetic diversity of female broods. Evidence exists in a few species to support the Red Queen hypothesis (e.g. [15, 32, 40, 46]), but this is a theme beyond the scope of the present paper.

In the present work we question how much direct evidence supports the maintenance of polyandry in the conditions presented by GDDRH. Moreover, the validity of the hypothesis beyond the original study group remains questionable. For this purpose, we conducted a literature survey to determine the number of studies that directly test GDDRH within eusocial Hymenopteran and non-Hymenopteran taxa. Next, we performed meta-analyses to test if polyandry (direct studies) or genetic diversity generated by other mechanisms (indirect studies) elicits lower parasite prevalence across taxa.

## Results

Of the 2106 studies found, 10 met the criteria of directly testing GDDRH and 32 were classified as indirect (Table 1).

**Table 1:** Direct and indirect tests of the ‘genetic diversity for disease resistance’ hypothesis based on the literature search terms “Polyandry” + “genetic diversity” + “disease resistance”

Author(s)	Year	Direct or Indirect	Species	Evidence for GDDRH
Shykoff and Schmid-Hempel^a^ [63]	1991	Indirect	Bumblebee (*Bombus terrestris*)	Yes
Shykoff and Schmid-Hempel^b^ [64]	1991	Indirect	Bumblebee (*Bombus terrestris*)	Yes
Liersch and Schmid-Hempel [39]	1998	Indirect	Bumblebee (*Bombus terrestris*)	Yes
Baer and Schmid-Hempel [5]	1999	Direct	Bumblebee (*Bombus terrestris*)	Yes
Coltman et al. [13]	1999	Indirect	Soay Sheep (*Ovis aries*)	Yes
Meagher [42]	1999	Indirect	Deer Mouse (*Peromyscus maniculatus*)	Yes
Schmid-Hempel & Crozier [59]	1999	Indirect	Phylogenetic Comparison	Mixed
Neumann and Moritz [47]	2000	Direct	Honeybee (*Apis mellifera*)	No
Baer and Schmid-Hempel [6]	2001	Direct	Bumblebee (*Bombus terrestris*)	Yes
Baer and Schmid-Hempel [7]	2003	Direct	Bumblebee (*Bombus terrestris*)	Yes
Carr et al. [11]	2003	Indirect	Monkey Flower (*Mimulus guttatus*)	Yes
Tarpy [69]	2003	Direct	Honeybee (*Apis mellifera*)	Yes*
Hughes and Boomsma [25]	2004	Indirect	Leaf Cutter Ant (*Acromyrmex echinatior*)	Mixed
Puurtinen et al. [54]	2004	Indirect	Freshwater Snail (*Lymnaea stagnalis*)	Yes
Pearman and Garner [52]	2005	Indirect	Frog (*Rana latastei*)	Yes
Calleri et al. [9]	2006	Indirect	Termite (*Zootermopsis angusticollis*)	Yes
Hughes and Boomsma [26]	2006	Indirect	Leaf Cutter Ant (*Acromyrmex echinatior*)	Yes
Tarpy and Seeley [70]	2006	Direct	Honeybee (*Apis mellifera*)	Yes
Field et al. [18]	2007	Indirect	Earthworm (*Lumbricus terrestris*)	No
Ross-Gillepsie et al. [57]	2007	Indirect	Naked Mole Rat (*Heterocephalus glaber*)	Yes
Seeley and Tarpy [60]	2007	Direct	Honeybee (*Apis mellifera*)	Yes
Altermatt and Ebert [2]	2008	Indirect	Freshwater Planktonic Crustacean (*Daphnia magna*)	Yes
Hughes et al. [25]	2008	Indirect	Meta analysis of eusocial Hymenoptera	Yes
Reber et al. [56]	2008	Indirect	Ant (*Formica selysi*)	Yes
Invernizzi et al. [29]	2009	Indirect	Honeybee (*Apis mellifera*	Yes
Jensen et al. [31]	2009	Indirect	Honeybees (*A. mellifera, A. mellifera carnica, A. m. ligustica,* and *A. m. mellifera*)	Yes
Lively [41]	2010	Indirect	Mathematical Model	Yes
Ugelvig et al. [73]	2010	Indirect	Ant (*Cardiocondyla obscurior*)	Yes
Ganz and Ebert [20]	2010	Indirect	Freshwater Planktonic Crustacean (*Daphnia magna*)	Yes
Allen et al. [1]	2011	Indirect	Fire Ant (*Solenopsis invicta*)	No
Vojvodic et al. [75]	2011	Indirect	Honeybee (*Apis mellifera*)	Yes
Whitehorn et al. [76]	2011	Indirect	Bumblebee (*Bombus muscorum*)	Yes
Bourgeois et al. [8]	2012	Indirect	Honeybee (*Apis mellifera*)	Yes
Franklin et al. [19]	2012	Direct	Western Tent Caterpillar (*Malacosoma californicum pluviale*)	No
Wilson-Rich et al. [79]	2012	Indirect	Honeybee (*Apis mellifera*)	No
Lee et al. [38]	2013	Indirect	Honeybee (*Apis mellifera*)	Yes
Whitehorn et al. [77]	2014	Indirect	Bumblebees (*Bombus muscorum* and *Bombus jonellus*)	Mixed
Desai and Currie [16]	2015	Direct	Honeybee (*Apis mellifera*)	Mixed
Simone-Finstrom et al. [66]	2016	Indirect	Honeybee (*Apis mellifera*)	Yes
Thonhauser et al. [72]	2016	Direct	House Mouse (*Mus musculus*)	No
Andras [4]	2017	Indirect	Sea Fan (*Gorgonia ventalaina*)	Yes
Saga et al. [58]	2020	Indirect	Wasp (*Vespula shidai*)	Yes

*Support for the hypothesis following a “bet-hedging” strategy hypothesis

Of the 10 direct studies identified, only two tested non-hymenopteran taxa. Of the eight publications that tested hymenopteran species, five found evidence that disease prevalence and promiscuity were negatively correlated (Table 1). One study found mixed results [16], while Tarpy [69] reported lower variance in parasite infection amongst colonies compared to single fathered broods, arguing these results support GDDRH as a bet-hedging strategy. Only one publication did not find evidence supporting GDDRH (Table 1). The two non-hymenopteran species in the direct studies data set utilized the house mouse (*Mus musculus*) and the western tent caterpillar (*Malacosoma californicum pluviale* [19, 72]; neither found evidence for decreased disease resistance in polyandrous relative to monandrous broods (Table 1).

**Fig. 1:**
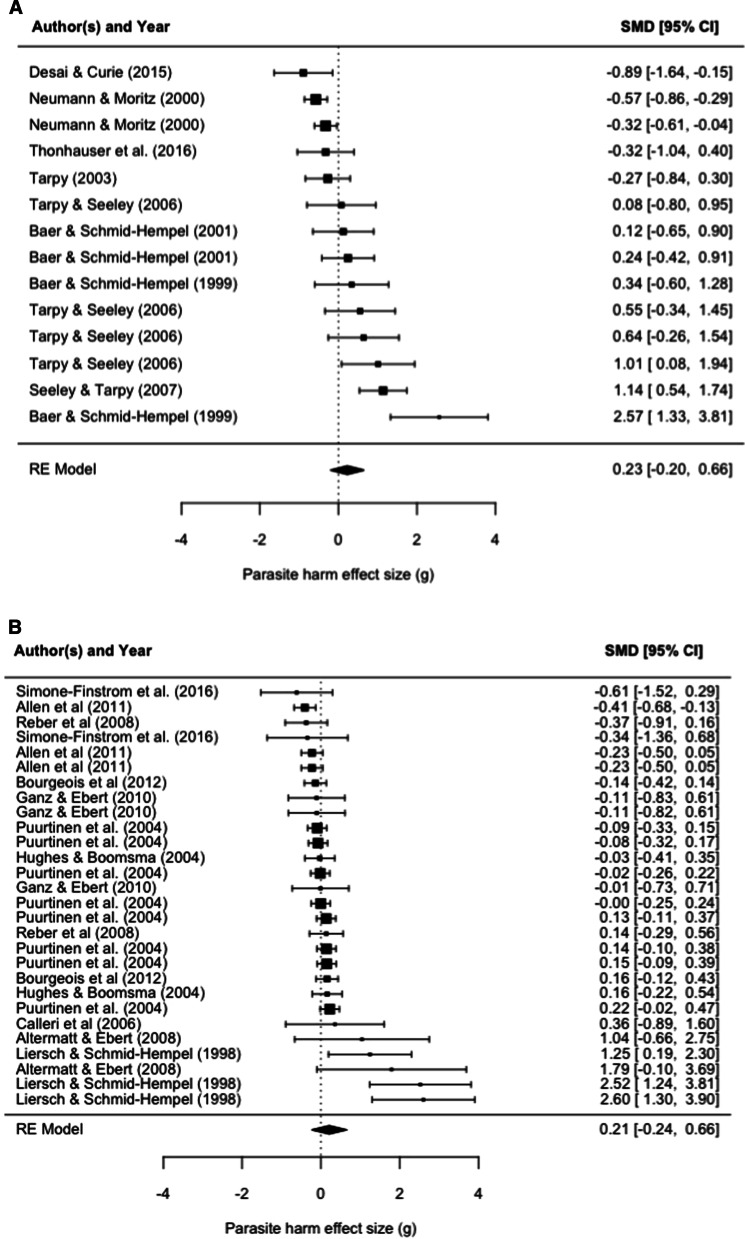
Forest plots of direct **A** and indirect **B** studies on the effects of host genetic diversity on parasite host harm effect size (*g*). Positive effect sizes show studies where parasite host harm is greater in low polyandrous groups whereas negative effect sizes show cases of greater host harm in high polyandrous host groups. The dotted line shows an effect size of zero (no relationship between diversity and parasite harm). The first y-axis shows the study the effect size was calculated from and the second y-axis shows the standard mean difference (SMD) calculation with confidence intervals. The size of the dot represents sample size

**Fig. 2:**
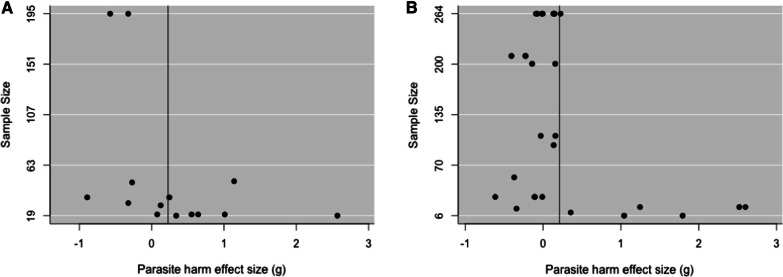
Funnel plots of direct **A** and indirect studies **B** data sets. Points on the graphs show the relationship between effect size and experiment sample size for each study. The vertical lines represent the effect size predicted by each meta-analysis model

**Fig. 3:**
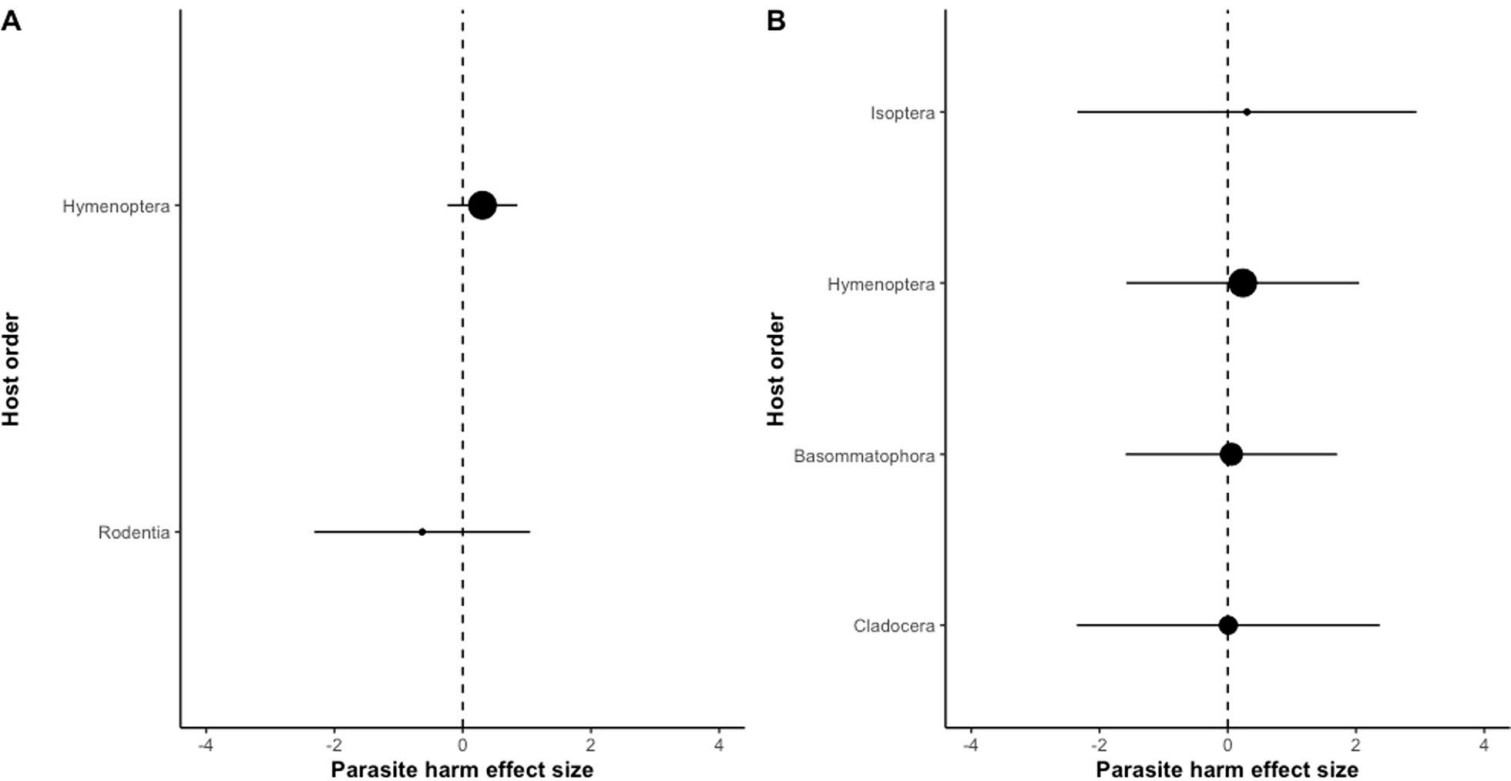
Parasite host harm effect size (*g*) for hosts grouped by “order” for direct **A** and indirect **B** studies. The size of the dot represents sample size

In total we extracted 14 effect sizes from 8 studies directly testing for GDDRH in our meta-analysis (Additional file 1: Table S1). When directly tested, polyandry increases parasite resistance (*g* = 0.2283, p < 0.0001, Fig. 1A). When separating host species by Order, we find that Hymenoptera (*g* = 0.3078) and Rodentia (*g* = -− 0.6314) did not significantly differ from each other (Q = 0.5401, p = 4624, Fig. 3A), suggesting that the GDDRH holds outside Hymenoptera. However, as this comparison is limited to two categories, where Rodentia consisted of 1 effect size, and with publication bias present (Fig. 2A), we cannot accept this result. Considering our meta-analysis results, based on a small data set, we conclude that the current available direct empirical evidence is insufficient in providing support for GDDRH outside of Hymenoptera.

In the second meta-analysis, we extracted 28 effect sizes from 10 studies indirectly testing GDDRH. Here, we also find that increased host population genetic diversity reduces parasite host harm (*g* = 0.21, p < 0.0001, Fig. 1B). Moderator analysis shows that host Orders Cladocera (*g* = 0.0076), Hymenoptera (*g* = 0.2345), Isoptera (*g* = 0.2991), and Basommatophora (*g* = 0.0563) do not significantly differ from each other (Q = 0.1183, p = 0.9896, Fig. 3A). However, we again find publication bias in this second data set, with effect sizes not falling symmetrically around the overall effect size (Fig. 2B), indicating that more studies are needed to properly test for GDDRH.

## Discussion

From our literature survey two patterns emerge: i) the number of empirical studies directly testing ‘genetic diversity for disease resistance’ hypothesis is surprisingly low and ii) eusocial Hymenoptera are the preferred study model in the direct studies data set.

The prevalence of eusocial Hymenoptera in direct studies is likely because of the characteristics that make the system very tractable in this type of research. The group is colonial and infections can be performed in the field or in the laboratory with the possibility of coinfection by multiple parasites. Moreover, artificial insemination is achievable, which allows the manipulation of mated males per female; this practice was carried out in some of the studies reported here imposing a mating system that does not naturally occur. For example, honeybees are not monandrous in nature, but two studies that were labeled as direct tests forced monandry through artificial insemination [60, 70].

Based on our first meta-analysis, we found no clear direct empirical evidence supporting GDDRH outside eusocial Hymenoptera. However, the outcome mostly reflects the very low number of studies directly testing the hypothesis outside the group (1 out of the 8 studies). On the other hand, the indirect studies encompassed a wider taxa diversity and the meta-analysis found support for reduced parasite load in genetically more diverse host populations. However, a funnel plot analysis confirms publication bias. Together our analyses stress the need for more studies testing the ‘genetic diversity’ hypothesis, and in particular its extension hypothesis GDDRH, namely outside eusocial Hymenoptera.

There could be ecological reasons why direct studies in eusocial Hymenoptera showed support for the GDDRH. Two aspects that could be important for disease transmission are dispersal and eusociality. Eusociality involves the formation of distinct behavioral castes. Typically, one to a few females reproduce with the rest of the colony members being made up by non-reproductive individuals [14]. When a female forms a colony, all offspring in the group have high levels of relatedness. Consequently, the colony could be quickly overtaken by a parasite that has efficiently evolved to overcome any resistance genotype of the group. In this scenario, there may be strong selection for the queen to mate multiply, increasing the level of genetic diversity within her colony. Conversely, when individuals of a population disperse, reducing contact with siblings (and often their own species), chances for parasite transmission are lower. Evolutionarily this could reduce pressure on the host species to evolve mechanisms that increase genetic diversity, including via polyandry. Although the vast majority of Hymenoptera are solitary, the species where direct evidence has been found for GDDRH are eusocial.

Future work should address this question specifically, i.e., if eusociality and density might be increasing the selection for polyandry. Namely, these studies should test if disease reduction also occurs within polyandrous broods from non-eusocial species with higher dispersal rates or with solitary lives. In these conditions disease transmission chances are lower and may result in relaxed selection for parasite resistance [43]. Utilization of a species that exhibits a mix of sociality may be particularly insightful. For example, *Ageniella (Lissagenia) flavipennis* is a spider wasp that exhibits both solitary nesting and colonies of cohabiting individuals [17].

Tarpy [69] observed a reduction in the variance of parasite load in polyandrous colonies compared to monandrous colonies. The authors argue that the results are consistent with polyandry as a ‘bet-hedging’ reproductive strategy [82]. Simply put, multiple paternity reduces the likelihood of severe population reduction by parasite load because distinct patrilines can show differences in susceptibility to parasite infection [61]. Long-term, i.e., between generations, polyandrous females reduce variance in offspring fitness relative to that of monandrous females [30]. Variation in disease resistance amongst patrilines of *A. mellifera* has been confirmed [8, 29], and argues in favor of this reasoning. However, our literature review of direct studies highlighted the lack of empirical evidence specifically addressing this topic. All studies in Table 1 have examined infection in one generation. Future multigenerational experimental evolution work could be used to invoke host-parasite coevolution under monandry and polyandry. This approach may also help to elucidate whether a threshold level of virulence is required for polyandry to be advantageous under host/parasite coevolution.

## Conclusions

Polyandry is ubiquitous throughout the animal taxa. Several hypotheses explaining the high levels of polyandry observed in some species of eusocial insects have been put forth and are reviewed in Oldroyd and Fewell [49]. While there could be many reasons for its prevalence in nature, parasites may be one strong selective force operating in the maintenance of polyandry. However, our analyses indicate that to better understand the exact conditions favoring polyandry in the terms proposed by GDDRH more direct studies are mandatory. The relationship between polyandry and the exact levels of genetic diversity in host populations required to increase infection resistance must be further teased apart [34]. Likewise, to understand if the GDDRH only applies to the Hymenoptera, greater research efforts directly testing GDDRH in non-Hymenoptera are necessary. Moreover, examining infection rates and polyandry in different social environments might shed some light on the interaction between different social structures, the evolution of polyandry, and disease resistance.

Continuing to investigate the ‘genetic diversity for disease resistance’ hypothesis would assist in understanding how, and in what taxa parasitic selection influences the evolution of polyandry. On the other hand, the generalized acceptation of the GDDRH may reflect the inflated influence of a few direct tests with strong positive findings in eusocial Hymenoptera. Also, probably contributing to the generalized acceptance of the theory is the high support found in the literature indirectly testing the hypothesis despite not reporting polyandry per se.

## Methods

For definition purposes, ‘parasites’ will be used to mean all infecting agents, which include microbial pathogens. We used the guidelines Preferred Reporting Items for Systematic reviews and Meta-Analysis (PRISMA) outlined in Moher et al. [44] to undergo a literature search to glean studies that test the GDDRH both directly and indirectly (see Additional file 2: Fig. S1). To determine how much direct evidence supporting the GDDRH exists, a literature search was conducted using both Google Scholar and Web of Science. Combinations of the keywords; “Polyandry”,”Genetic Diversity”, “Multiple Mating”, and”Disease Resistance” were used for each database in May 2020. Next, citations were removed if replicates were found between searches and databases, as well as non-peer reviewed sources (i.e. books, dissertations). This resulted in 2106 citations being left. Titles were then evaluated for relevance and 2018 papers were removed. Of the articles remaining we assessed the following parameters: (1) disease or infection evaluation (2) genetic diversity alterations and (3) determination of study type (reviews were eliminated). Those studies that assessed infection, and altered genetic diversity were further evaluated and parsed into direct and indirect studies.

This process resulted in 42 studies that were grouped into direct or indirect tests of the GDDRH (Table 1). For the purposes of this paper, a direct study testing GDDRH is defined as one that i) directly measures parasitic infection of offspring, with infection performed either in the field or direct infection in the laboratory, and ii) incorporated correlation with high vs. low genetically diverse host populations through mating strategy differences, i.e. monandry vs. polyandry. Comparing monandrous broods to polyandrous broods are valuable treatments when genetic relatedness is not available: monandry should have lower levels of genetic diversity providing the best alternative to polyandry for comparison. However, one study does not use monandrous broods, but rather compares the relationship between parasite load and varying levels of promiscuity (i.e., 10 to 28 mated males per queen in Neumann & Moritz, 2000, with genetic relatedness reported). Another study manipulated male genetic diversity that led to the effective mating rate being 1.3 versus 4 males [5]. Both studies were classified as direct tests of the GDDRH. Indirect studies examine parasitic infection in groups that may have different levels of genetic diversity generated through other mechanisms. For example, genetic diversity was manipulated through groups founded by one (monogyne) or more than one female (polygyne). Although that is a mechanism for increasing genetic diversity, it does not address polyandrous behavior, and as a result those studies were classified as indirect as long as infection was also assessed.

We then assessed the studies to determine if they could be included in the meta-analysis based on the following parameters: (i) a comparison between high and low genetic diversity groups and (ii) assess parasite success (i.e., mortality). We excluded studies that used heterogeneity as a measure for genetic diversity as we were interested in the benefits of polyandry at the population level and not individual level on parasite success. We also excluded studies that were mathematical models and meta-analyses. This left 8 direct studies and 10 indirect studies that we gleaned data from to conduct our meta-analysis.

We conducted a meta-analysis following the methods described in Hedges [24], using Hedges’s *g* to estimate effect sizes. Standard mean difference was calculated using the *escalc* function in the package *metafor* in R v. 1.3.1056 (R Development Core Team). The web-based tool WebPlotDigitizer (https://automeris.io/WebPlotDigitizer/userManual.pdf) was used to extract data from publication plots when raw data was not available.

The terms of the GDDRH posit that polyandry is favored when it results in increased genetic diversity, gambling in the likelihood that half-siblings will vary in resilience to parasites. In the direct studies dataset, standard mean difference effect sizes were calculated by extracting parasite harm mean measurements and their standard deviations in two groups: monandry or low polyandry and high polyandry. In one study [70], *t*-values and degrees of freedom were extracted due to the lack of means and standard deviations. As most direct studies looked at the effects of GDDRH in Hymenoptera, we first performed a nested random mixed effects model using the *rma.mv* function to account for phylogenetic non-independence. The same method to obtain standard mean difference was applied on studies indirectly testing GDDRH; however, here, groups were categorized into low genetic diversity and high genetic diversity.

Additionally, we tested whether the magnitude of the relationship was dependent on eusocial Hymenoptera for both datasets by performing a third analysis using “host Order” as a moderator variable.

Last, we tested for a potential publication bias by plotting a funnel plot for both datasets, i.e., direct and indirect studies.

## Supplementary Information

**Additional file 1: Table S1.** The left column includes the citations used to make Fig. 1. The number of data points taken from each paper is in the right column (N = 14 for direct; N = 28 for indirect). 

**Additional file 2:** **Figure S1.** Prisma Analysis decision tree.

## Data Availability

Data is available via Dryad and can be accessed here: https://doi.org/10.5061/dryad.dv41ns1zw.

## Abbreviation

GDDRH: Genetic Diversity for Disease Resistance Hypothesis
