# Supplementary material for: Direct evidence for increased disease resistance in polyandrous broods exists only in eusocial Hymenoptera
Source: BMC Ecol Evol. 2021 Oct 20;21:189. doi: 10.1186/s12862-021-01925-3 (PMC8527725; doi:10.1186/s12862-021-01925-3)
Supplement: Supplementary file 1 — Additional file 1: Table S1. The left column includes the citations used to make Fig. 1. The number of data points taken from each paper is in the right column (N = 14 for direct; N = 28 for indirect). [file 12862_2021_1925_MOESM1_ESM.docx]

Table S1: The left column includes the citations used to make figure 1. The number of data points taken from each paper is in the right column (N=14 for direct; N=28 for indirect).

| **Citation** | **Direct or Indirect** | **Number of Effect sizes** |
| --- | --- | --- |
| Liersch and Schmid-Hempel 1998 | Indirect | 3 |
| Baer and Schmid-Hempel 1999 | Direct | 2 |
| Neumann and Moritz 2000 | Direct | 2 |
| Baer and Schmid-Hempel 2001 | Direct | 2 |
| Puurtinen et al. 2001 | Indirect | 8 |
| Tarpy 2003 | Direct | 1 |
| Hughes and Boomsma 2004 | Indirect | 2 |
| Calleri et al. 2006 | Indirect | 1 |
| Tarpy and Seeley 2006 | Direct | 4 |
| Seeley and Tarpy 2007 | Direct | 1 |
| Altermatt and Ebert 2008 | Indirect | 2 |
| Reber et al. 2008 | Indirect | 2 |
| Ganz and Ebert 2010 | Indirect | 3 |
| Allen et al. 2011 | Indirect | 3 |
| Bourgeois et al. 2012 | Indirect | 2 |
| Desai and Curie 2015 | Direct | 1 |
| Simone-Finstrom et al. 2016 | Indirect | 2 |
| Thonhauser et al. 2016 | Direct | 1 |
