# Supplementary figures and images for: Direct evidence for increased disease resistance in polyandrous broods exists only in eusocial Hymenoptera
Source: BMC Ecol Evol. 2021 Oct 20;21:189. doi: 10.1186/s12862-021-01925-3 (PMC8527725; doi:10.1186/s12862-021-01925-3)

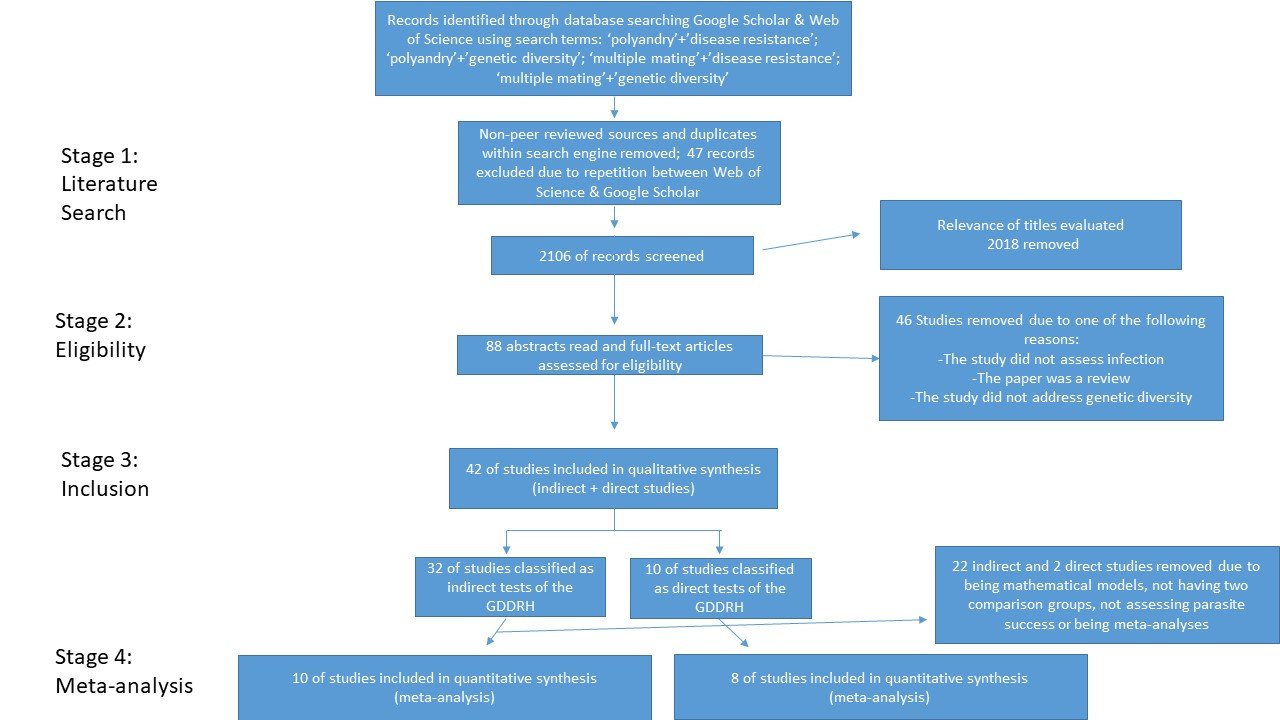

Supplement: Supplementary file 2 — Additional file 2: Figure S1. Prisma Analysis decision tree. [file 12862_2021_1925_MOESM2_ESM.jpg]
